# The Role of Initial Oligomers in Amyloid Fibril Formation by Human Stefin B

**DOI:** 10.3390/ijms140918362

**Published:** 2013-09-05

**Authors:** Ajda Taler-Verčič, Tiina Kirsipuu, Merlin Friedemann, Andra Noormägi, Mira Polajnar, Julia Smirnova, Magda Tušek Žnidarič, Matjaž Žganec, Miha Škarabot, Andrej Vilfan, Rosemary A. Staniforth, Peep Palumaa, Eva Žerovnik

**Affiliations:** 1 Department of Biochemistry, Molecular and Structural Biology, Jožef Stefan Institute, Ljubljana 1000, Slovenia; E-Mails: ajda.taler@ijs.si (A.T.-V.); mira.polajnar@ijs.si (M.P.); matjaz.zganec@ijs.si (M.Ž.); 2 Jožef Stefan’s International Postgraduate School (IPS), Ljubljana 1000, Slovenia; 3 Department of Gene Technology, Tallinn University of Technology, Tallinn 12618, Estonia; E-Mails: tiina.kumm@ttu.ee (T.K.); merfrie@gmail.com (M.F.); andra.noormagi@ttu.ee (A.N.); jussmirnova@gmail.com (J.S.); 4 Department of Biotechnology and System Biology, National Institute of Biology, Ljubljana 1000, Slovenia; E-Mail: magda.tusek.znidaric@nib.si; 5 Faculty of Mathematics and Physics, University of Ljubljana, Ljubljana 1000, Slovenia; 6 Department of Condensed Matter Physics, Jožef Stefan Institute, Ljubljana 1000, Slovenia; E-Mails: miha.skarabot@ijs.si (M.Š.); andrej.vilfan@ijs.si (A.V.); 7 Department of Molecular Biology and Biotechnology, University of Sheffield, Sheffield S10 2TN, UK; E-Mail: r.a.staniforth@sheffield.ac.uk

**Keywords:** human stefin B, cystatins, protein aggregation, prefibrillar oligomers, mechanism of amyloid fibril formation, ESI MS, SEC, lag phase

## Abstract

Oligomers are commonly observed intermediates at the initial stages of amyloid fibril formation. They are toxic to neurons and cause decrease in neural transmission and long-term potentiation. We describe an *in vitro* study of the initial steps in amyloid fibril formation by human stefin B, which proved to be a good model system. Due to relative stability of the initial oligomers of stefin B, electrospray ionization mass spectrometry (ESI MS) could be applied in addition to size exclusion chromatography (SEC). These two techniques enabled us to separate and detect distinguished oligomers from the monomers: dimers, trimers, tetramers, up to decamers. The amyloid fibril formation process was followed at different pH and temperatures, including such conditions where the process was slow enough to detect the initial oligomeric species at the very beginning of the lag phase and those at the end of the lag phase. Taking into account the results of the lower-order oligomers transformations early in the process, we were able to propose an improved model for the stefin B fibril formation.

## 1. Introduction

Misfolding, aggregation and amyloid fibril formation of proteins are common underlying features of amyloidoses, both systemic and local, including several major neurodegenerative diseases. Beside classical amyloidogenic peptides and proteins like amyloid-β peptide, α-synuclein and Cu/Zn-SOD-1, many other amyloidogenic proteins exist, including stefins, which do not cause a known amyloid pathology, yet are prone to aggregate *in vitro* and in cells. Stefin B, together with stefin A and some cathepsins have been identified in the core of amyloid plaques of various origins [1], nevertheless amyloid plaques containing only stefins or cathepsins have not been found.

Stefins are 11 kDa intracellular proteins [2,3], which are ubiquitously expressed in human tissues [4]. They belong to the family of cystatins [5,6], which are endogenous cysteine protease inhibitors specific for the papain-family of cysteine proteinases [3] and are classified as the IH clan in the MEROPS scheme [7].

Stefin B is a globular protein of 98 amino acid residues which does not contain disulphide bonds (it has one cysteine at site 3) or carbohydrate groups [5]. Stefins are globular α/β-proteins [3] composed from a β-pleated-sheet structure, consisting of four longer and one shorter antiparallel β-strand and form an 18-residue long α-helix [8]. Several structural data is already available including monomer [8], dimer [9], tetramer [10] structures and even a model for fibrils [11]. As said, the protein is evenly distributed among different cells and tissues [12]. In the cell, it is localized in the cytoplasm, lysosomes, and also in the nucleus [13].

Mutations in the cystatin B gene, coding the protein human stefin B, are the cause for progressive myoclonus epilepsy of type 1 (EPM1), known as Unverricht-Lundborg disease [14–16]. By majority the EPM1 mutations lead to decreased protein expression because of dodecamer repeats in the non-coding region. However, some missense mutations were also reported that cause functional and structural changes to the protein, which—as we proposed in 2005—may aggregate in cells and *in vivo* [17]. In accordance with loss of function and gain in toxic function, EPM1 patients show features of increased oxidative stress and neurodegeneration [1,18].

Several studies of stefins and their mutant stability, mechanisms of folding and fibrillogenesis [19–25] have demonstrated that stefin B is an amyloidogenic protein and can serve as a suitable model for studies of amyloid fibril formation [26–28], amyloid-membrane interactions [29–31] and amyloid-induced cytotoxicity [32,33].

Indeed, experimental conditions used here under which stefin B forms amyloid fibrils are not physiological. In the case of model proteins, the physiological conditions are not thus important, because the reaction rate can be slowed or increased at choice (more easily than for amyloid-β peptide), which in turn can contribute to more detailed information about the whole process. Initial stages of protein aggregation to amyloid fibrils often involve transitions between oligomers during the lag phase. The prefibrillar oligomers are thought to be the toxic entities and it is important to determine, which oligomer exerts toxicity and what its conformational state is [34].

Some characteristics of the prefibrillar oligomers of stefin B have been described in previous studies [29,32,33]. The prefibrillar oligomers form nuclei of nanometer sizes, comprising at least eight or more monomers [32]. In the case of stefin B, the globules accumulating in the lag phase at pH 3.3 were approximated from 16-mer to 32-mers [32] and in the mathematically simulated model of the fibrillation at pH 4.8, even 64-mers [35]. The role of lower-order oligomers in the fibrillation of stefin B, whether they are on or off-pathway, is not completely clear at this moment. In the model for the mechanism of stefin B fibril formation [35], it has been proposed that lower-order oligomers, such as tetramers, might be off-pathway, as judged from an unusual behavior at high protein concentrations. As many different model proteins were used to study the process of amyloid fibrillation, several models have been proposed, although they have some common features like off-pathway oligomers [36–38], and variable toxicity of different prefibrillar species [36,39].

Electrospray ionization mass spectrometry (ESI MS) is a powerful technique for identification of oligomeric states of proteins even in heterogeneous multicomponent systems. ESI is a soft ionization technique and it has been successfully applied for detection of noncovalent protein-protein interactions and for studies of protein complexes [40–42]. Intermolecular noncovalent interactions are responsible for the aggregation of folded polypeptide chains into multimers, determining protein complexes quaternary structure [43]. In fact, if higher-order oligomers are stable, the ESI MS can give the precise information about their oligomeric states. ESI MS generates a distribution of multiply charged molecular ions ([M + zH^+^]^z+^), which depends on the conformational state of the protein. Conformational changes are evident from the shifts in charge-state distribution of protein ions in ESI MS spectra, and rely on the well-confirmed assumption that in the electrospray ionization process, proteins with compact conformations produce lower charge-state ions than proteins with more open conformations [44]. Charge-state distribution analysis in ESI MS has been extensively used for evaluation of large scale protein conformational changes [45–50].

Previously, ESI MS has been used for evaluation of oligomers and prefibrillar aggregates of various peptides and proteins [51], such as, for example, amyloid-β peptide fragment 10–30 [52], nucleobase-functionalized beta-peptides [53], insulin [54], transthyretins [36,55,56], human beta-2-microglobulin [57], native hemocyanins [58,59], flavoproteins [60], bifunctional kinase/phosphatase [58]. We have previously used ESI MS for the determination of the complex between oligomers, tetramers, and dimers of stefin B and monomers of amyloid-β peptide [42].

Commonly, protein tetramers [55], hexamers [52,58] or octamers [60] have been observed, but higher-order oligomers like 18-mers [59,61] have also been detected. In most of the studies thus far, mainly even-numbered oligomers have been described, whereas in some cases, odd-numbered oligomers have been reported to occur [57]. For example, in the case of beta-2-microglobulin trimers, pentamers, heptamers and nonamers have been detected [62], and in the case of amyloid-β peptide, even more odd-numbered oligomers have been detected from trimers, pentamers, heptamers, nonamers, 11-mers and up to 13-mers [63]. So far, ESI MS has not been used to determine the oligomeric profile and conformation of stefin B during the lag phase of its amyloid-like fibrillation process.

The aim of the current study was to apply size exclusion chromatography (SEC) and ESI MS to characterize the early oligomeric states of stefin B preceding amyloid-fibril formation to improve the model for the fibril formation. The fibrillation was started from higher-order oligomeric, tetrameric, dimeric and monomeric states of stefin B and the time course of the early transitions and equilibrium distribution between the oligomers was monitored.

## 2. Results and Discussion

Recombinant stefin B forms well-defined oligomers, which have been detected using various methods, such as SEC [32,64,65], transmission electron microscopy (TEM) [32,65], atomic force microscopy (AFM) [32,65] and dynamic light scattering (DLS) [32,33]. We have focused here on transitions between oligomers at the beginning and at the very end of the lag phase in order to improve the model of the stefin B fibrillation process [35]. Despite the fact that most of amyloidogenic proteins’ oligomers are not very stable, stefin B oligomers are stable enough to be observed with SEC and ESI MS, which may arise from domain-swapped dimerization [9,10,32], which is a characteristic feature of cystatins. It was shown that domain-swapped dimers of stefin A are more stable than the monomers [66]. Moreover, stefin B tetramer is composed of two domain-swapped dimers [10].

### 2.1. Fibril Formation by Stefin B; ThT Fluorescence, TEM and AFM Observations

**Figure 1:**
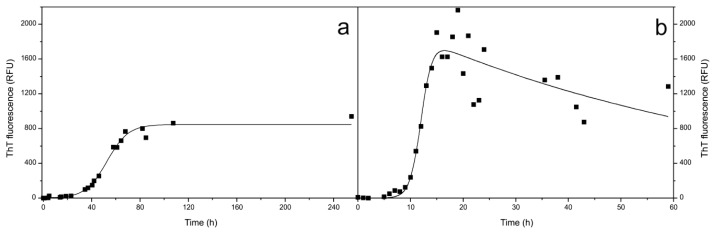
Stefin B fibril formation as monitored by ThT fluorescence. ThT fluorescence was monitored at pH 4.8 and room temperature (**a**) and pH 7.5 and 50 °C (**b**). At certain time points, aliquots were taken and fluorescence was measured. At the plateau of the reactions, aliquots were taken for TEM and AFM.

We have already obtained many kinetic data on amyloid fibril formation by stefin B, its EPM1 mutants and also some other point mutations [19,21,26–28,35,67,68]. Here, we applied two conditions which both lead to mature fibrils but they differ in the length of the lag phase and total amount of fibrils at the plateau of the process. Fibril formation at pH 4.8 and room temperature (Figure 1a) is four times slower than the process at pH 7.5 and 50 °C (Figure 1b).

**Figure 2:**
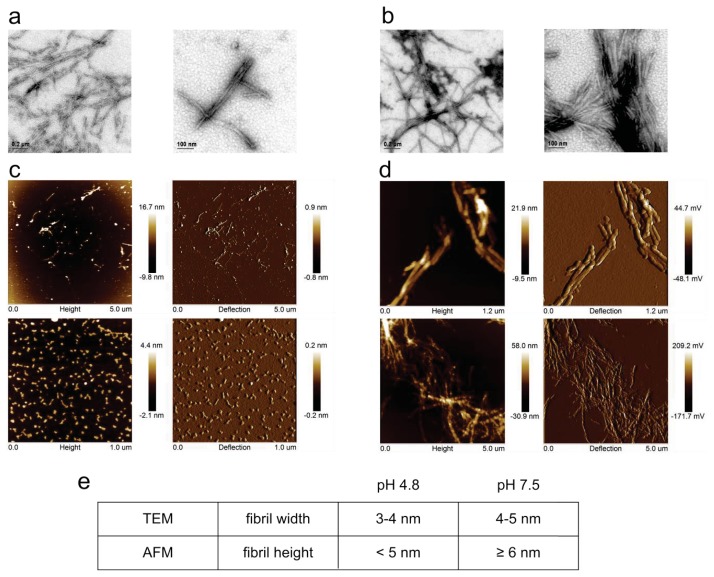
TEM and AFM images of stefin B fibrils. TEM images of stefin B fibrils at pH 4.8, room temperature (**a**) and pH 7.5, 50 °C (**b**); and AFM images of stefin B fibrils at pH 4.8, room temperature (**c**); and pH 7.5, 50 °C (**d**) are shown at two different magnifications for each sample. For each AFM measurement, height and deflection are shown. Fibril width and height are summarized in the table (**e**).

We have also analyzed the morphology of the mature fibrils using TEM and AFM under both conditions (Figure 2). At pH 4.8 in the plateau phase, many fibrils have grown (Figure 2a), although many oligomers are also present as observed by AFM (Figure 2c). Fibrils are shorter than previously observed [35,68], which is likely because we applied constant mixing (which was never done before and it speeds up the process). At pH 7.5 and 50 °C in the plateau phase more fibrils can be seen, which is evident from ThT fluorescence (Figure 1b) as well as from both imaging techniques (Figure 2b,d). We also have noticed that some protein aggregates at pH 7.5 during the incubation time appeared.

Fibril width and height are summarized in the table in Figure 2e, where average values of many measurements done with each technique are given. Looking at these values, which seem 1nm lower at pH 4.8 than pH 7.5, it has to be taken into account that we are near the detection limit of TEM with an error of around 1 nm, whereas AFM gives values which are precise within 0.5 nm. Therefore, a tentative conclusion that fibrils of double height (6 nm) would grow at pH 7.5 cannot be made.

As it can be seen from the table in Figure 2e, a conclusion is met that fibrils at both conditions do not differ significantly in size or morphology, which is in agreement with an explanation that at both conditions dimers are the likely building blocks of the fibrils, although monomers also appeared in the ESI MS spectra at pH 7.5, 50 °C (Figure 9). We have also tried to obtain fibrils at pH 5.8, but at the time of observation no fibril growth was observed. Nevertheless, we concluded that the first 6 h at pH 7.5 and 50 °C the lag phase is nearly completed (results at 6 h present the very end of the lag phase) and at pH 5.8 and room temperature, we are at the beginning of the lag phase, far away from mature fibrils (which we assumed they would eventually form as they did at pH 4.8 and room temperature after 1.5 months [27]).

### 2.2. Stefin B Oligomers

**Figure 3:**
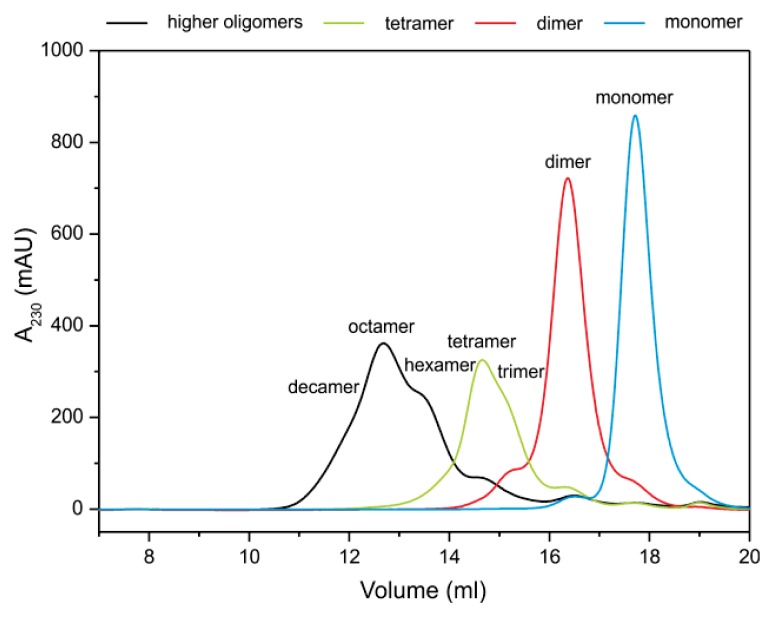
SEC of stefin B oligomers. Monomers, dimers, tetramers and higher-order oligomers were isolated from the mixture by Superdex™ 75 column and then loaded to the Superdex™ 200 column. Peaks were further analyzed by ESI MS. All four chromatograms are overlaid in one graph.

Separation of stefin B oligomers at pH 7.0 by SEC (Superdex™ 75) has been reported before by Ceru *et al.* [32], where monomers, dimers, tetramers and higher-order oligomers were SEC-purified and their morphology was observed by AFM. Higher-order oligomers eluted as a broad peak comprising hexamers, octamers, dodecamers, hexadecamers and even higher-order species, and were well separated from tetramers, dimers and monomers, which was also confirmed by DLS [32]. In this work, a more efficient separation and purification of the oligomers by SEC was obtained by consequent separations on Superdex™ 75 and Superdex™ 200 columns (Figure 3).

Monomers, dimers, tetramers and higher-order oligomers are well separated and in addition, on the right-hand side of the tetramer peak a shoulder of presumably trimers can be seen. The trimeric and hexameric shoulders were not observed previously [32].

**Figure 4:**
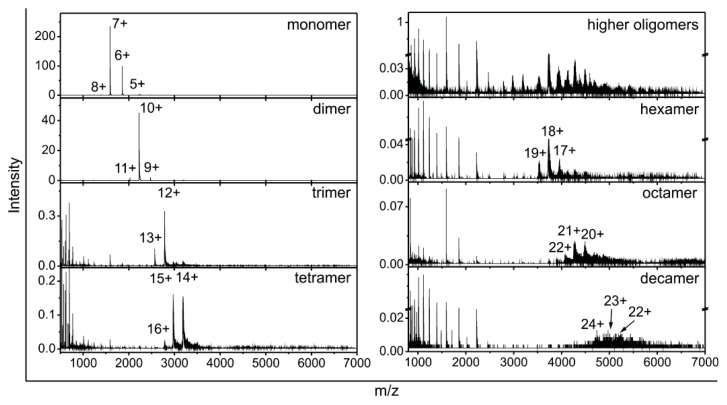
ESI MS of stefin B oligomers. SEC fractions containing stefin B oligomers were injected to ESI MS instrument and mass spectra corresponding to monomers, separated oligomers (dimers, trimers, tetramers, hexamers, octamers and decamers) and unseparated sample of all the higher-order oligomers were recorded. The numbers on the top of the peaks denote the charge states of the protein ions.

Isolated fractions corresponding to the peak maxima and the shoulders (Figure 3) were further analyzed by ESI MS. In Figure 4, ESI MS spectra are shown for the SEC-purified monomers and oligomers from dimers up to decamers. The total fraction of all higher-order oligomers (not fractionated with Superdex™ 200) is also shown. Those oligomers are stable at 4 °C and pH 7.0 for several weeks and only partial equilibration takes place.

Well defined ions characteristic for stefin B monomoners, dimers, trimers and tetramers can be seen. This is the first time that trimeric stefin B has been observed and characterized *in vitro*, whereas in cells the occurrence of the trimers apart from other oligomers was demonstrated before. In the same study, it has also been reported before that stefin B polymers grow by monomer addition *in vivo*, which can explain trimer formation [69]. Available structural data did not offer any solution for the trimer existence. Resolved tetramer is made of two domain-swapped dimers [10] and resolved dimer is also domain swapped [9].

In the ESI MS spectrum of the higher-order oligomers fraction, several peaks corresponding to different oligomers can be seen; however, their identification is rather difficult. To obtain more detailed information, different fractions of the broad peak collected from Superdex™ 200 column (Figure 3) were analyzed with ESI MS (Figure 4). ESI MS spectrum from the fraction of the right shoulder of the higher-order oligomers peak corresponds to hexamers, while the spectrum from the top of the peak corresponds to octamers (Figure 4).

We observed that for trimers and all oligomers higher than trimers, a small peak for monomer appears in the spectra, which could be some small in-source dissociation.

According to Figures 3 and 4, the presence of even higher-order oligomers instead of decamers could be presumed, but because of the very low ESI MS spectrum resolution and intensity, clear identification of these was not possible.

In the ESI MS spectra of stefin B oligomeric forms (Figure 4) the average charge of the ions increases and the charge-state distribution is shifted to higher m/z with increasing oligomeric state of the protein; at the same time the intensity of the ions decreases by hundreds.

### 2.3. pH Dependent Transitions Between Oligomers

**Figure 5:**
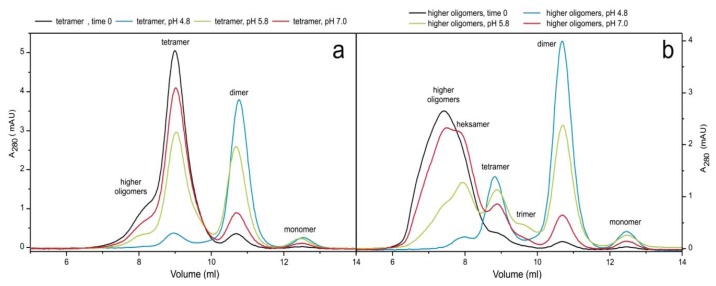
pH dependent transitions between stefin B oligomers. Samples were incubated for two hours at 23 °C at three different pH values (4.8, 5.8 and 7.0) and then analyzed by SEC. Transitions are shown for tetramers (**a**) and higher-order oligomers (**b**).

SEC and ESI MS were used to follow the initial time course of the transitions between oligomers and monomers at different pH values. Figure 5 shows the pH effect on transitions after two hours of incubation at 23 °C. The same experiment was done with addition of 9% (*v*/*v*) TFE (2,2,2,-trifluoroethanol) at room temperature and without the addition of TFE at 40 °C (not shown). TFE has the same partially denaturing effect on the protein structure as higher temperature (40 °C). Transitions were started from the separated tetramers and higher-order oligomers. It can be seen (Figure 5) that at pH 7.0 the transitions are slow, whereas at pH 4.8 transitions into dimers are almost completed in two hours. At pH 5.8 the same transitions take place with a medium rate.

When started from the tetramers (Figure 5a), the dissociation into dimers is seen, decreasing in rate with increasing pH. When started from the higher-order oligomers (Figure 5b), first a visible shift towards hexamers occurs and an additional peak of trimers appears. Those two peaks are not seen in the initial SEC-purified fractions of the oligomers.

pH has a strong effect on the oligomer transitions. The lower the pH, the stronger is the effect. At pH 4.8 the reaction—dissociation into dimeric form—is nearly completed in 2 h of observation. At pH 5.8 the transitions are of medium rate and proved most appropriate in order to observe different species on the way to dimer by SEC and ESI MS. Despite the fact that separated oligomers are stable at 4 °C and pH 7.0 for several weeks, when changing the temperature to 25 °C and adding TFE, higher-order oligomers and tetramers equilibrate slowly towards dimers, even at pH 7.0.

### 2.4. Time Course of the Transitions between Oligomers at pH 5.8 at Room Temperature Monitored by SEC and ESI MS—the Beginning of the Lag Phase

**Figure 6:**
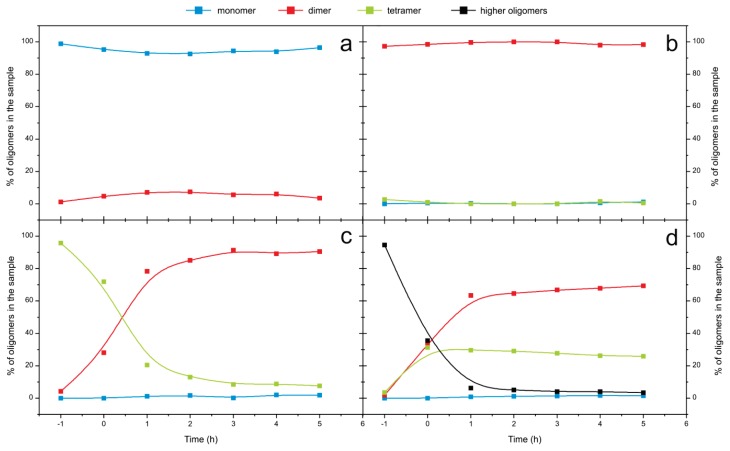
Time course of the transitions between stefin B oligomers at pH 5.8 and 25 °C followed by SEC. SEC-purified oligomers were incubated at pH 5.8, every hour an aliquot was analyzed by SEC. Curves were integrated and % for each oligomer content at every time point was calculated. Time marked as −1 is showing SEC-purified oligomers before the certain buffer was added. Processes were started from SEC-purified monomers (**a**); dimers (**b**); tetramers (**c**) and higher-order oligomers (**d**).

Transitions between different oligomers starting from the SEC-purified monomers, dimers, tetramers and higher-order oligomers were monitored using SEC (Figure 6). No changes in amounts of monomers and dimers were observed, whereas tetramers changed into dimers completely in 5 h and there were no further changes in 48 h (not shown). Dissociation speed of the tetramers is 1 × 10^−4^ s^−1^. Higher-order oligomers dissociated into tetramers and dimers in 1–2 h, and after that in 48 h (not shown), only a small decrease in the amount of tetramers was detected.

**Figure 7:**
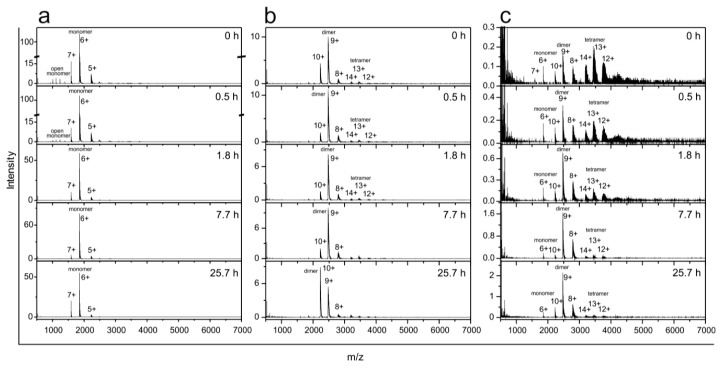
Time course of the transitions between stefin B oligomers at pH 5.8 and 23 °C monitored by ESI MS. SEC-purified oligomers were incubated at pH 5.8 without salt. At certain time points, aliquots were taken and analyzed by ESI MS. Numbers on the top of the peaks denote the charge states of protein ions. Transitions were monitored for SEC-purified monomers (**a**); dimers (**b**) and tetramers (**c**).

The same process—starting from the SEC-purified monomers, dimers and tetramers—was monitored also by ESI MS (Figure 7). During 25 h of observation no changes in the amount of monomers were observed (Figure 7a); whereas the charge state distribution of dimers changed (Figure 7b), which means that rearrangement of the surface charge occurred (average charge state changed from *q_av_* = 9.2 at *t* = 0 to *q_av_* = 9.6 at *t* = 2 after 25.7 h, which may present a partial opening of the protein conformation). Tetramers disappeared almost completely and dimers appeared instead (Figure 7c).

These slow and subtle conformational changes over longer times (>24 h) are only detectable by ESI MS, likely because SEC does not reach sufficient resolution power.

Thus, combined SEC and ESI MS results have confirmed that at the onset of amyloid fibril formation of stefin B (at pH below 6.0 and at room temperature) the tetramers and higher-order oligomers dissociate into dimers. We assume that these dimers are domain-swapped and are therefore, very stable. Higher-order oligomers are less stable than the tetramers and dissociate first into hexamers, tetramers and trimers, before dissociating completely into the dimers (Figure 5). SEC-purified monomers stay stable throughout (at pH 5.8), as do the SEC-purified dimers. Towards the end of the lag phase (lag phase is always defined based on ThT fluorescence (Figure 1)), as shown at pH 5.8 on Figures 6 and 7, monomers and dimers remain as the most likely building blocks of the prefibrillar oligomers and fibrils.

### 2.5. Time Course of the Transitions between Oligomers at pH 7.5 and 50 °C Monitored by SEC and ESI MS—the Whole Lag Phase

**Figure 8:**
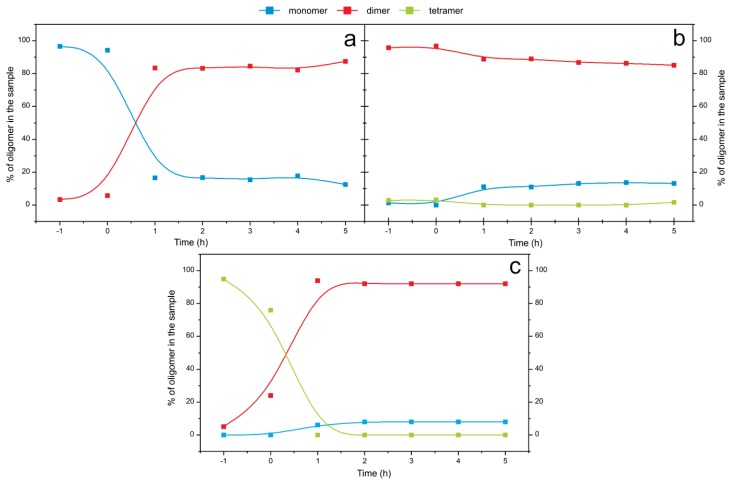
Time course of the transitions between oligomers at pH 7.5 and 50 °C monitored by SEC. Separated oligomers were incubated at pH 7.5, 50 °C; every hour an aliquot was analyzed by SEC. Curves were integrated and % for each oligomer content at every time point was calculated. Time marked as −1 is showing SEC-purified oligomers before the certain buffer was added. Processes were started from monomers (**a**); dimers (**b**) and tetramers (**c**).

Even though at pH 7.5 the transitions between oligomers were slow at room temperature, raising the temperature to 50 °C accelerated the transitions between oligomers and further led to amyloid fibril formation. The time course of the transitions between oligomers starting from the SEC-purified monomers, dimers and tetramers were firstly monitored by SEC (Figure 8). In the first 2 h at pH 7.5 and 50 °C, monomers changed into dimers; nevertheless, 15% of monomers still remained (Figure 8a). In the case of dimers, only 15% of dimers changed into monomers in the same 2 h (Figure 8b) and a small amount of the tetramers also remained in the sample. Tetramers dissociated into 92% of dimers and 8% of monomers (Figure 8c). Changes between the dimeric and monomeric forms of the protein indicate that there is equilibrium between the two species. The time of the observation is equal to the lag phase of the process under these conditions; after 6 h of incubation at pH 7.5 and 50 °C the fibril growth actually starts (Figure 1).

**Figure 9:**
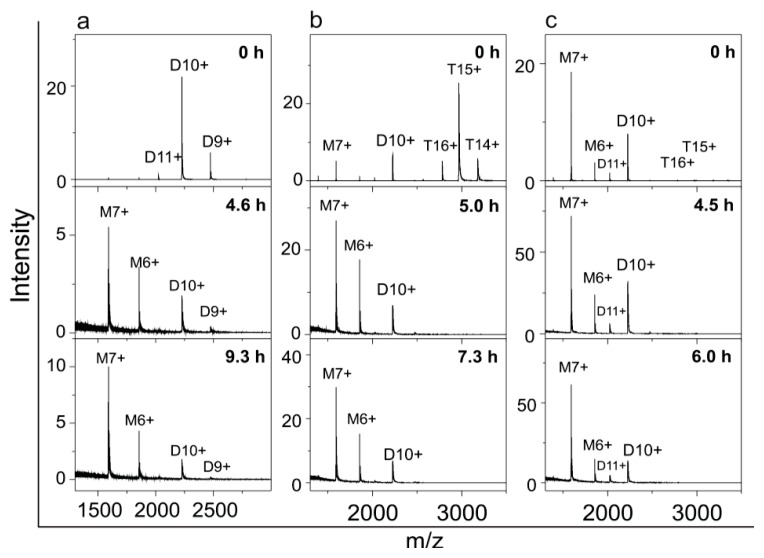
Time course of the transitions between oligomers at pH 7.5 and 50 °C monitored by ESI MS. Separated oligomers were incubated at pH 7.5 and 50 °C. At certain time points aliquots were taken and analyzed by ESI MS. Numbers on the top of the peaks denote the charge states of protein ions. Processes were started from SEC-purified dimers (**a**); tetramers (**b**) and higher-order oligomers (**c**).

Secondly, ESI MS samples, starting from the SEC-purified dimers, tetramers and higher-order oligomers, were taken at different time points during the fibrillation process and in all samples dimers and monomers increased while peaks corresponding to higher-order species diminished (Figure 9). This is in agreement with SEC results (Figure 8), where during the process time some monomers remained or appeared in the sample. From the ESI MS experiment it may seem that monomers are the dominant species (Figure 9). We think that monomers are less abundant species (Figure 8), and that this effect is due to the fact that smaller molecules yield better signals in ESI MS spectra.

### 2.6. Model for the Mechanism of Amyloid Fibril Formation by Stefin B

Stefin B already proved to be a good model for amyloid fibril formation [11,26–28,35,68,70]. Fibrillation is slow enough to allow detailed studies of the mechanism and on the other hand the outcome of the process is the same as for other amyloidogenic proteins (protofibrils and mature fibrils) involved in various amyloidoses.

In a previous study, we proposed a model for stefin B fibril formation based on concentration and temperature dependencies for the process. Three energies of activation were defined: first-ordered process nucleation (E_a_ of 55 ± 4 kcal/mol), second-ordered process fibril growth (E_a_ of 27 ± 5 kcal/mol) and off-pathway oligomeric state, which was also called nonproductive oligomerization (E_a_ of 95 ± 5 kcal/mol at 35 °C) [35].

In order to obtain more data to improve the previous fibrillation model, concentration dependency of the process during the lag phase was studied using SEC (Table 1). Fibrillation was performed, both, at pH 5.8, 25 °C and pH 7.5, 50 °C. Once again, the concentration dependent experiments showed that at the end of the observation time, the dimers are the prevalent species, and in addition, some monomers remain or appear in the samples.

**Figure 10:**
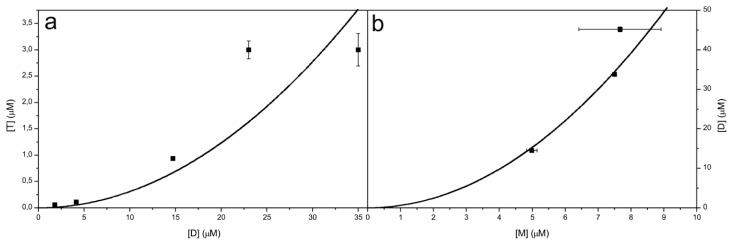
Equilibrium between dimer [D] and tetramer [T] (pH 5.8, 25 °C) (**a**) and monomer [M] and dimer [D] (pH 7.5, 50 °C) (**b**). Fitted to square function, according to a second order reaction ([T] = 0.00308 μM^−1^[D]^2^ and [D] = 0.612 μM^−1^[M]^2^).

Two equilibrium constants were calculated from these data (Table 1, Figure 10). At pH 7.5, 50 °C monomers and dimers are in equilibrium; *K*_d_ (*K*_d_ = [M]^2^/[D]) of the process is 1.63 ± 0.31 μM. Transitions between monomers and dimers are fitted to square function according to a second order reaction. *K*_d_ (*K*_d_ = [D]^2^/[T]) for the tetramer formation is 325 ± 180 μM. Transitions between dimers and tetramers are fitted to square function according to a second order reaction.

Škerget *et al.* [35] proposed the mechanism of amyloid fibril formation based on concentration and temperature dependence studies. The mechanism showed that the process starts from the monomer, and continues through the dimers, with an off-pathway connection to dimers and tetramers, whereas the main pathway proceeds via an oligomeric nucleus and leads to fibrils. We used this model and focused on the transitions of lower-order oligomers in the lag phase, further clarifying what species may be off-pathway. Tetramers are not always on the path from higher-order oligomers to dimers. Higher-order oligomers are reversible as we observed when freezing to −80 °C and thawing the sample, which resulted in an equilibrium, shifted towards higher-order oligomers.

The dissociation of part of a dimer to a monomer at pH 7.5 and 50 °C (Figure 8) indicates that there is a kind of monomer in equilibrium with the dimer. Because it was shown that domain-swapped dimers are the building blocks of fibrils [11], we termed this monomer in equilibrium the open monomer (in domain-swapped dimers, the basic fold of monomers is preserved [9,10], the structure of the monomer has to open substantially before domain swapping into the dimer can occur).

Based on the NMR protection pattern [11], it was concluded that not only 3D domain-swapped dimers are responsible for the initiation of amyloid fibril growth; what is more, domain-swapped dimers with the loop between strands four and five as in the tetramer position could be at the core of stefin B fibrils. It was also shown that monomers are relatively frequently available for solvent exchange [11], which is in agreement with our result, where monomers are in equilibrium with dimers during the fibrillation process.

Lowering the pH to 4.7 and adding 10% TFE affects the structure of both stefin B monomers and dimers, which was shown by NMR [70]. Shown changes are similar to those conformational changes between the monomer [8] and tetramer [10] in the crystal structure. Actually, four different types of dimers have been observed in solution and are likely due to *cis-trans* proline isomerization (stefin B has five proline residues), which is also responsible for the conformation of the loop between strands four and five [70]. Those different dimer conformations in solution could also explain our results; where at pH 7.5 and 50 °C 15% of monomers stay or appear in the solution, which means that only one dimer population is in equilibrium with monomers. It could also be suggested that from only one of those dimers the fibril formation can actually start. One of the species is in equilibrium with the tetramer and one of them results from higher-order oligomers when they dissociate, which also explains why fibril formation started from higher-order oligomers has shorter lag phase (data not shown) than when started from other lower-order oligomers: dimers or tetramers.

Stefin B cytotoxicity was extensively studied earlier [29–33]. Monomers, dimers and tetramers were found to be not cytotoxic [32], the toxicity started with higher-order oligomers (hexamers, dodecamers, *etc.*) [32]. Previous studies have also shown that toxicity is most likely related to amyloid-membrane interactions, which could result in membrane perforation [32]. Wild-type protein, which is a mixture of different oligomers is similarly able to form pores in planar lipid bilayers and those pores are cation selective, but are not lethal to the cells [31]. It also has been shown that stefin B higher-order oligomers are inserted into membranes more effectively than other lower-order oligomers or monomers [32]. There are no significant differences in the cytotoxicity between the higher-order oligomers and acid induced prefibrillar oligomers (pH 3 and pH 5) which accumulate in the lag phase of the reaction [32,33]. The conformation of the toxic species of amyloidogenic proteins is predicted to be at least partially unfolded or even molten globule [33].

In conclusion, in previous studies based on the NMR determined model of the structure for the stefin B mature fibrils [11] and toxicity studies [29–33], the off-pathway higher-order oligomers (except the tetramers and one kind of dimers) and prefibrillar oligomers have been found to be toxic to cells. From the present study, we can additionally conclude that for all fibril formation processes induced by different conditions, prefibrillar oligomeric fraction, formed from one form of the dimers (presumably domain-swapped) is common. It can be assumed that the building block of the higher-order oligomers, which are also toxic, has the same form as the dimer.

## 3. Experimental Section

### 3.1. Expression and Purification of Recombinant Stefin B

Recombinant human stefin B (S3E31) was used for all studies. Protein inserted in pET11a vector was expressed in BL21 pLysS (DE3) cells. Expression was induced with IPTG (1 mM final concentration). After induction, cells grew for 3 h. Cells were lysed and resuspended in lysis buffer (50 mM TRIS/HCl, pH 8.0, 0.1 M NaCl, 1 mM EDTA). Sample was homogenized with ultraturax; DNA and some other proteins were precipitated with 4% (*w*/*v*) polyethyleneimine. Stefin B was further purified using affinity chromatography (elution from papain sepharose with triethylamine, pH 10.65) and size exclusion chromatography (Superose 200 and 0.01 M phosphate buffer, pH 6.1, 0.12 M NaCl). Proteins were then stored at −20 °C in 0.01 M phosphate buffer, pH 6.1, 0.06 M NaCl. The recombinant proteins have Ser at position 3 instead of Cys to prevent covalent disulfide bond formation, which anyway does not occur *in vivo*.

### 3.2. Size Exclusion Chromatography (SEC on FPLC)

On the Superdex™ 75 column (10 × 300 mm) (Amersham Biosciences, Uppsala, Sweden), connected to FPLC (Amersham Biosciences, Uppsala, Sweden) stefin B elutes as a set of well-defined oligomers, allowing isolation of monomers, dimers, tetramers and higher-order oligomers. SEC was performed at room temperature. Flow was 0.5 mL/min. 500 μL of protein (~3 mg/mL) were applied to the column. 0.01 M phosphate buffer, pH 7.0, 0.15 M NaCl was standard buffer, only when preparing samples for ESI MS 0.02 M ammonium acetate, pH 7.5 was used. We isolated different oligomers by loading them to Superdex™ 75 column and concentrate them with Amicons ultra 3 kDa (Millipore, Billerica, MA, USA) before usage.

Samples for ESI MS were prepared using Superdex™ 200 GL column (10 × 300 mm) (Amersham Biosciences, Uppsala, Sweden) connected to GE AKTA Purifier system (Amersham Biosciences, Uppsala, Sweden). The flow rate was 0.75 mL/min. Sample volume was 100 μL and 0.2 mL fractions were collected.

### 3.3. Electrospray Ionization Mass Spectrometry (ESI MS) Studies of Stefin B

Selected fractions from SEC (stefin B in 0.02 M ammonium acetate, pH 7.5) were injected directly into the ESI-Q-TOF mass spectrometer (QStar ELITE, Applied Biosystems, Foster City, CA, USA) by a syringe pump at the flow rate of 8 μL/min (sample volume was 50 μL). ESI MS spectra were recorded in the positive mode in region 500–7000 *m*/*z* using following instrument parameters: ion spray voltage 5500 V; source gas 45 L/min; curtain gas 20 L/min; declustering potential 60 V; focusing potential 320 V; detector voltage 2350 V. The obtained spectra were deconvoluted by program Bioanalyst 2.0 (Applied Biosystems, Foster City, CA, USA). The resolution of QStar ELITE (Applied Biosystems, Foster City, CA, USA) for a 6000 Da protein is 11,000.

### 3.4. Monitoring Fibrillation of Stefin B by Thioflavin T (ThT) Fluorescence

Thirty four micromolar protein was incubated at pH 4.8 (0.1 M sodium acetate, 0.15 M NaCl, 9% (*v*/*v*) TFE, 25 °C, 300 rpm) and pH 7.5 (0.02 M ammonium acetate, 0.15 M NaCl, 9% (*v*/*v*) TFE, 50 °C, 300 rpm). At certain time points, 50 μL aliquots were taken, 570 μL of ThT with absorbance 0.66 at 416 nm were added and fluorescence was measured (exc. 450 nm; emis. 482 nm).

### 3.5. Transmission Electron Microscopy (TEM)

Two different samples (at pH 4.8 (0.1 M sodium acetate, 0.15 M NaCl, 9% (*v*/*v*) TFE, 25 °C, 300 rpm) and pH 7.5 (0.02 M ammonium acetate, 0.15 M NaCl, 9% (*v*/*v*) TFE, 50 °C, 300 rpm)) were taken at the plateau of the process (~95 h for both samples). Protein concentration was 34 μM. 20 μL of solution, diluted 10–50 times as appropriate, were applied to a formvar and carbon coated grid. After 5 min the samples were soaked away and the grid was stained with 1% uranyl acetate.

Philips CM 100 transmission electron microscope was used operating at 80 or 100 kV, magnifications were from ×10,000 to ×130,000. Images were recorded by a Gatan Bioscan CCD camera, using Digital Micrograph software (version 3.3; Gatan Inc., Pleasanton, Washington DC, WA, USA, 1998).

### 3.6. Atomic Force Microscopy (AFM)

Two different samples at pH 4.8 (0.1 M sodium acetate, 0.15 M NaCl, 9% (*v*/*v*) TFE, 25 °C, 300 rpm) and pH 7.5 (0.02 M ammonium acetate, 0.15 M NaCl, 9% (*v*/*v*) TFE, 50 °C, 300 rpm) were taken at the plateau of the reaction (~38 h (pH 7.5), ~200 h (pH 4.8)). Protein concentration was 34 μM. 15 μL of each sample were applied onto common mica which was cleaved earlier with sellotape to have an ultraflat surface. After 10 min, samples were washed away with 1 mL of water and mica was dried with nitrogen flow.

Images were recorded with Nanoscope IIIa (Digital Instruments, Santa Barbara, CA, USA), scanner E in contact mode (Veeco MSNL probe, tip C, *k* = 0.01 N/m) and tapping mode (Olympus OMCL-AC160TS, k ~30 N/m, f ~320 kHz) in ambient air.

### 3.7. Monitoring Oligomer Transitions by SEC

Superdex™ 75 column (10 × 300 mm) (Amersham Biosciences, Uppsala, Sweden), connected to FPLC (Amersham Biosciences, Uppsala, Sweden) was used with the constant flow 0.5 mL/min at room temperature. Column was equilibrated with 0.01 M phosphate buffer, pH 7.0, 0.15 M NaCl. Fifty microliters of sample were applied on the column. Afterwards, the peaks were integrated and ratio between different oligomers was calculated. As control we used isolated oligomers which were >95% pure.

Oligomers were incubated in two different buffers at pH 5.8 (0.1 M phosphate buffer, 0.15 M NaCl, 9% (*v*/*v*) TFE, 25 °C) and 7.5 (0.02 M ammonium acetate, 0.15 M NaCl, 9% (*v*/*v*) TFE, 50 °C, 300 rpm). Every hour an aliquot of 50 μL was analyzed by SEC.

For pH dependent oligomer transformations, three different buffers were used: 0.1 M phosphate buffer, pH 7.0, 0.15 M NaCl, 9% (*v*/*v*) TFE; 0.1 M phosphate buffer, pH 5.8, 0.15 M NaCl, 9% (*v*/*v*) TFE and 0.1 M sodium acetate, pH 4.8, 0.15 M NaCl, 9% (*v*/*v*) TFE. Protein concentration was 34 μM. Samples were incubated 2 h at 23 °C. Results obtained at 40 °C and with no TFE added were very similar and are not shown.

For concentration dependent oligomer transformations, two different buffers were used: 0.02 M ammonium acetate, pH 7.5, 0.15 M NaCl, 9% (*v*/*v*) TFE, 50 °C, 300 rpm and 0.1 M phosphate buffer, pH 5.8, 0.15 M NaCl, 9% (*v*/*v*) TFE, 25 °C, 300 rpm. Samples were incubated at defined conditions for five hours. Every hour an aliquot was taken and analyzed by SEC. Protein concentrations were 4, 9, 34, 75 and 100 μM at pH 5.8 and 34, 75 and 100 μM at pH 7.5. Also, the control samples (before changing conditions) were analyzed by SEC. Reactions were started from the oligomer mixture (wild type protein after purification). Differences in the certain oligomers content in the control are due to sample concentration (when concentrating sample, the ratio between oligomers was changing).

### 3.8. Monitoring Oligomer Transitions by ESI MS

SEC-purified oligomers were incubated at pH 5.8 (0.02 M ammonium acetate, 9% (*v*/*v*) TFE, 23 °C) and pH 7.5 (0.02 M ammonium acetate, 50 °C in a quartz cell with continuous agitation in the presence of 2 μM ThT). Protein concentration was 30 μM. At different time points, aliquots were taken, filtered and analyzed by ESI MS. Average charge state (*q_av_*) was computed as follows:

(1) $$q_{av}=\frac{\sum_{i=1}^{n}q_{i}\times w_{i}}{\sum_{i=1}^{n}w_{i}}$$

where *n* is the number of observed charge states in a mass spectrum, *q_i_* is the net charge of the *i* charge state, and *w_i_* is the signal intensity of the *i* charge state.

## 4. Conclusions

In order to understand the role of protein aggregates in the pathogenesis of neurodegenerative diseases, it is necessary to study molecular mechanisms of amyloid fibril formation and the contribution of each oligomeric intermediate, to that process.

Such knowledge will contribute to the development of effective new strategies for treatment of a growing number of patients with most common kinds of dementias, such as Alzheimer’s and Parkinson’s disease, and other rarer diseases affecting the brain due to the same phenomenon of protein aggregation.

In this work, we have shown that ESI MS in conjunction with SEC is a useful technique to monitor both the oligomeric states of stefin B at equilibrium and the time course of transitions preceding amyloid fibril formation *in vitro*.

This is the first time that trimer of stefin B has been detected *in vitro*, and furthermore, it was not detected as mixture but as an isolated peak (Figures 3, 4 and 5b).

We have also confirmed that partial denaturation by TFE has the same effect on oligomer transformations as partial denaturation by higher temperature (40 °C).

Different conditions like pH, temperature, and ionic strength in the case of stefin B all lead to the same result at the end of the process—In all cases fibrils do not differ significantly.

The new results (a proportion of the monomer present during the fibrillation process, different dimer conformations in equilibrium, and transitions into dimers (presumably domain-swapped) towards the end of the lag phase) are consistent with previous studies [11,70].

**Figure 11:**
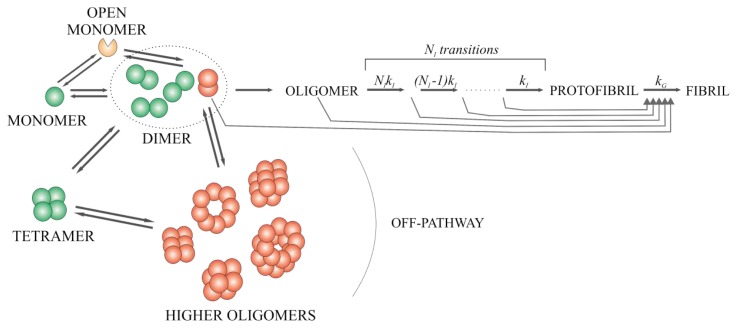
Improved model for the mechanism of amyloid fibril formation by stefin B. The kinetic scheme is based on previous studies [10,28,35,68] and the present results. Off-pathway explanation has been confirmed and improved. Non-toxic species are colored green, toxic are colored red and potentially toxic are colored orange.

Taking into account the already published (described) and the present results, we were able to improve the model for stefin B amyloid fibril formation [35] further clarifying the transitions between different oligomers from dimers, tetramers to higher-order oligomers, at the beginning and at the very end of the lag phase (Figure 11).

We believe that stefin B is a good model protein as it has certain advantages over other amyloidogenic proteins (for example, its rather stable oligomers, which can be easily SEC-purified and studied). Thus, this research provides valuable information for a deeper understanding of the mechanism of amyloid fibril formation and some generalization to other proteins can be made.

## Figures and Tables

**Table 1:** Concentration dependent transitions between oligomers at pH 5.8, 25 °C and pH 7.5, 50 °C studied by SEC. Fibrillation was monitored during 5 h with 1 h intervals. Numbers denote % content of the certain oligomers in the sample. M states for monomers, D for dimers, T for tetramers and H for higher-order oligomers.

	Protein concentration (μM)	Amount of oligomers in the sample (%) (time –1—beginning of the experiment, before changing the conditions)				Amount of oligomers in the sample (%) (time 5 h—end of the experiment)			
	
		M	D	T	H	M	D	T	H
pH 5.8	4	5	56	28	11	4	89	7	–
	9	5	56	28	11	4	91	5	–
	34	5	56	28	11	3	86	11	–
	75	20	53	21	6	23	58	16	3
	100	20	53	21	6	20	66	12	2

pH 7.5	34	20	53	21	6	15	85	–	–
	75	20	53	21	6	10	90	–	–
	100	8	66	21	5	9	89	2	–
